# Timing of first bath in term healthy newborns: A systematic review

**DOI:** 10.7189/jogh.12.12004

**Published:** 2022-08-17

**Authors:** Mayank Priyadarshi, Bharathi Balachander, Shuchita Gupta, Mari Jeeva Sankar

**Affiliations:** 1 Department of Neonatology, All India Institute of Medical Sciences, Rishikesh, Uttarakhand, India; 2 Department of Neonatology, St. Johns Medical College Hospital, Bangalore, Karnataka, India; 3 World Health Organization, Geneva, Switzerland; 4 Department of Pediatrics, All India Institute of Medical Sciences, New Delhi, India

## Abstract

**Background:**

This systematic review of intervention trials and observational studies assessed the effect of delaying the first bath for at least 24 hours after birth, compared to conducting it within the first 24 hours, in term healthy newborns.

**Methods:**

We searched MEDLINE via PubMed, Cochrane CENTRAL, Embase, CINAHL (updated till November 2021), and clinical trials databases and reference lists of retrieved articles. Key outcomes were neonatal mortality, systemic infections, hypothermia, hypoglycaemia, and exclusive breastfeeding (EBF) rates. Two authors separately evaluated the risk of bias, extracted data, and synthesized effect estimates using relative risk (RR) or odds ratio (OR). The GRADE approach was used to assess the certainty of evidence.

**Results:**

We included 16 studies (two trials and 14 observational studies) involving 39 020 term or near-term healthy newborns. Delayed and early baths were defined variably in the studies, most commonly as >24 hours (six studies) and as ≤6 hours (12 studies), respectively. We performed a post-hoc analysis for studies that defined early bath as ≤6 hours. Low certainty evidence suggested that bathing the newborn 24 hours after birth might reduce the risk of infant mortality (OR = 0.46, 95% confidence interval (CI) = 0.28 to 0.77; one study, 789 participants) and neonatal hypothermia (OR = 0.50, 95% CI = 0.28-0.88; one study, 660 newborns), compared to bathing within first 24 hours. The evidence on the effect on EBF at discharge was very uncertain. Delayed bath beyond 6 hours (at or after nine, 12, or 24 hours) after birth compared to that within 6 hours might reduce the risk of hypothermia (OR = 0.47, 95% CI = 0.36-0.61; four studies, 2711 newborns) and hypoglycaemia (OR = 0.39, 95% CI = 0.23-0.66; three studies, 2775 newborns) and improve the incidence of EBF at discharge (OR = 1.12, 95% CI = 1.08-1.34; six studies, 6768 newborns); the evidence of the effect on neonatal mortality was very uncertain.

**Conclusion:**

Delayed first bath for at least 24 hours may reduce infant mortality and hypothermia. Delayed bath for at least 6 hours may prevent hypothermia and hypoglycaemia and improve EBF rates at discharge. However, most of these conclusions are limited by low certainty evidence.

**Registration:**

PROSPERO 2020 CRD42020177430.

Newborn bathing is practised in many contexts on the day of birth as is thought to eliminate pollutants from the skin and prevent infection. However, this practice is based on cultural beliefs rather than evidence [1]. There are various methods of bathing neonates like tub bathing, sponge bathing, swaddled bathing, and under running-water bathing [2]. The two commonly used methods in hospitals include washing the neonate with a wet cloth (sponge-bath) or immersion of the body in tubs (tub-bath). Immersion bath has been shown to decrease heat loss in neonates, thereby decreasing the predisposition to hypothermia [3].

Bathing is a stressful procedure for a neonate and an early first bath has been shown to destabilize the vitals in apparently healthy neonates, especially temperature, glucose levels, and respiratory status [4,5]. Depending on severity, hypothermia can lead to poor activity, lethargy, and difficulty feeding which further predisposes the neonate to hypoglycaemia. Other consequences of the first bath may include tachypnoea, an increase in pulmonary vascular resistance, hypoxia, and pulmonary haemorrhage, secondary to hypothermia. Moreover, the timing of the first bath may be crucial for establishing breastfeeding and improving exclusive breastfeeding (EBF) rates. In many hospitals, the first bath is given soon after birth, separating the neonate from the mother, depriving her/him of the opportunity of skin-to-skin contact (SSC) with the mother, which can potentially hamper breastfeeding rates [6].

The World Health Organization (WHO) recommends delaying bathing until 24 hours (h) after birth, and when not possible, to be delayed for at least 6 h [7]. However, this recommendation was based on expert consensus. Delaying the first bath may allow time for a neonate’s vitals to stabilize after birth. A pilot study showed that delaying the first bath until 24 h of life was associated with benefits from vernix caseosa on the skin and adequate time for SSC with the mother’s participation in her child’s bathing [8]. Improved SSC with the mother may improve breastfeeding rates, body temperatures, and blood glucose levels in neonates [6].

There is currently no evidence-based recommendation for the timing of the first bath in healthy neonates. The objective of this review was to determine the impact of a first bath delayed for at least 24 h, compared to an early bath within the first 24 h, on neonatal mortality, hypothermia, hypoglycaemia, and exclusive breastfeeding rates in term healthy newborns.

## METHODS

Randomized controlled trials (RCTs) including cluster randomized trials or quasi-randomized trials in human neonates were eligible for this review. If the number of RCTs was found to be inadequate (<3) or the optimal information size was not met, we included observational studies (before-after/cohort/case-control/cross-sectional study analysed like case-control). The study population were term neonates (up to 28 completed days of life). We excluded the studies if most of the participants (at least 50%) were either low birth weight or preterm neonates. Studies were included if delayed first bath (after 24 h of age) was compared to early first bath (within 24 h of age) in neonates. The outcomes of interest were neonatal mortality (all-cause death in the first 28 days of life), systemic infections (sepsis, pneumonia, or possible serious bacterial infection), any respiratory morbidity (respiratory distress, pulmonary hemorrhage, or pulmonary hypertension), hypothermia (recorded temperature less than 36.5°C or 97.7°F), hypoglycaemia (recorded glucose level less than 2.5 mmol/L or 45 mg/dL), timing of breastfeeding initiation and exclusive breastfeeding rate at discharge and at 6 months of age.

### Search methodology

The databases were searched independently by two authors (MP and BB). The search was conducted in the following databases: MEDLINE (1966 onwards) via PubMed, Cochrane Central Register of Controlled Trials (CENTRAL, The Cochrane Library), Embase (1947 onwards), and CINAHL (1981 onwards). We conducted the first search till March 31, 2020, which was later updated till November 30, 2021. Searches were limited to human studies. There were no language or publication date restrictions. We also searched related conference proceedings (eg, Pediatric Academic Societies abstracts), clinical trial registries (eg, clinicaltrials.gov), and the reference list of all trials/studies identified. We contacted experts or researchers in the field, if necessary, for information on unpublished and ongoing trials. The search strategy is provided in Appendix S1 in **Online Supplementary Document**.

### Data extraction and management

Two authors (MP and BB) extracted data independently using a pilot-tested data collection form to collect information on study setting, design, methods, participants, intervention (eg, timing and type of bath, duration, and the person implementing intervention), co-interventions (eg, use of warmer after bath), outcomes, and treatment effects from each included study. If data from study reports were insufficient, we contacted study authors to request further information or any clarifications, if required. Additional data was obtained by personal communication from the author for one study [9].

### Assessment of risk of bias in included studies

Two authors (MP and BB) independently assessed the methodological quality of the selected trials/ studies. Quality assessment was undertaken using the Cochrane Risk of bias (RoB 2.0) tool for randomized trials, and using the ROBINS-I tool for observational studies [10,11]. Any disagreements between the authors were resolved by discussion.

### Statistical analysis

Meta-analysis was performed using Stata 15.1 (StataCorp, College Station, TX, USA). Pooled estimates for categorical outcomes were calculated from the relative risk (RR) or odds ratios (OR) and their 95% confidence intervals (CIs) by the generic inverse variance method. We used the adjusted RR and OR from the studies where available. We used intention-to-treat analysis to calculate effect sizes for studies that provided the number of participants non-adherent to intervention with separate outcome data for these participants. We examined heterogeneity between study results by inspecting the forest plots and quantifying the impact of heterogeneity using the *I^2^* statistic. We pooled the results of individual studies using the fixed-effect model if *I^2^* was ≤60%. If we detected significant heterogeneity (*I^2^*>60%), we explored the possible causes by examining the population, intervention, outcome, and settings. If there was significant clinical heterogeneity, we used subgroup analyses or deferred from pooling the study results. If there was no clinical heterogeneity, we used the random-effects model for meta-analysis. We used the GRADEpro software for assigning the quality of evidence [12].

## RESULTS

**Figure 1:**
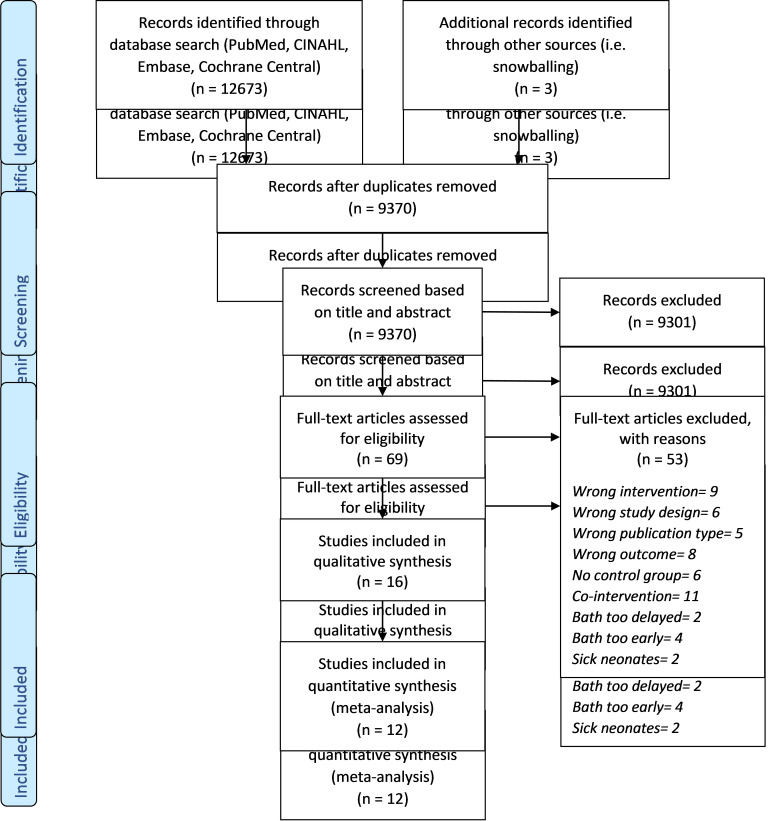
PRISMA flowchart depicting the selection of studies included in the review.

We included 16 studies in the review, 12 of which were included for meta-analysis (**Figure 1**, **Table 1**). The reasons for the exclusion of four studies from meta-analysis and their results have been summarized in Appendix S2 in **Online Supplementary Document**.

**Table 1:** Characteristics of the studies included in the review

S.No.	Study author, year	Setting (level) / LMIC or HIC	Study design	Study population/Mean BW/ gestation	Intervention/ post-intervention cohort details	Control/ pre-intervention cohort details	Outcome parameters of interest	Results	Comments
1.	Anderson 2021 [13]	Military health system (4 medical centers and 5 community hospitals), USA; HIC	Before after study	Term newborns (≥37 wk gestation) born within the nine facilities. Exclusion: Preterm newborns, those admitted to a higher level of care (NICU), newborns born at home, or those requiring significant resuscitation. n = 900 (post-intervention = 450, pre-intervention = 450), BW, gest = not mentioned.	Baths performed in parent’s room after 24 h of life; parents were instructed to tub bathe their newborn and encouraged to complete the bath in <5 min; newborn placed on prewarmed radiant warmer after bath to towel dry Post-intervention period: not mentioned	Sponge bath at 2 to 4 h of life. Pre-intervention period: not mentioned.	Incidence of hypothermia (axillary temp <36.5 C) post-bath; temp records at 30 min, 60 min, 120 min of life and 8 h till discharge.	Significant lower incidence of post-bath hypothermia (*P* < 0.001); fewer hypothermic temperature readings from birth until 8 h of life.	Exact timing of post-bath temp assessment not clear; use of warmer post-bath in delayed bath cohort but not mentioned in early bath cohort; immersion bath in the delayed bath cohort vs sponge bath in early bath cohort (tub bath causes less heat loss).
2.	Brennan 2020 [14]	Community hospital mother-baby unit, USA; HIC	Before after study	Included population: Term newborns. Exclusion: not mentioned. n = not mentioned. BW, gest = not mentioned.	Delayed-immersion swaddle bathing (mean time = 16 h). Post-intervention period = not mentioned.	Current bathing practice (mean time = 4.6 h). Pre-intervention period = not mentioned.	Hypothermia, hypoglycaemia, and breastfeeding rate at discharge (definitions not mentioned).	Mean temperature of newborns after bath. Increased post-implementation, from 98.2°F to 98.5°F; no significant difference in exclusive breastfeeding at discharge.	Full text not available, data extracted from abstract; number of participants not mentioned, hence could not be included in meta-analysis.
3.	Chamberlain 2018 [15]	Midwestern health system, USA; HIC	Before after study	Newborns born without complications or need for specialized care. Exclusion criteria: Newborns who were transferred to a higher level of care due to prematurity and/or physical distress. n = 660 (post-intervention = 330, pre-intervention = 330). BW, gest = not mentioned.	Bath delayed until 24 h after birth unless contraindicated by a bloodborne pathogen due to risk of transmission of disease from mother's blood or neonate's vernix. Post-intervention period = charts from June 2017 – May 2018.	Bathed <24 h of life. Pre-intervention period: charts from January – December 2014.	Exclusive breastfeeding (EBF) rates, temperature and glucose stability, and percentage of weight loss post-intervention.	Significant decrease in the amount of blood glucose levels equal or below 45 (*P* = 0.001) and cold stress (*P* < 01) post-intervention. No change in exclusive breastfeeding rates at discharge or percentage weight loss.	Data for hypoglycemia not in extractable form; adherence to intervention not noted; outcome definition for EBF missing.
4.	DiCioccio 2018 [16]	Family maternal unit in a tertiary care community hospital, USA; HIC	Before after study	Neonates born at 35 0/7 wk gestation or later. Exclusion criteria: newborns admitted to the NICU within the first 12 or more h of life, newborns discharged from the NICU, and newborns separated from mothers (eg, maternal transfer to a higher-acuity setting). n = 996 (post-intervention = 548, pre-intervention = 448). Gest = not mentioned/BW = 3.3 kg.	Delaying the first newborn bath until at least 12 h after birth (median timing = 17.9 h). Post-intervention period = July – August 2016	Early bathing at 2 h of birth (median timing: 1.9 h). Pre-intervention period: January – February 2015.	In-hospital EBF rates and plans to use human milk at discharge.	Increased in-hospital EBF rate from 59.8% before the intervention to 68.2% after the intervention (*P* = 0.006). Increase in use of human milk as a part of the discharge feeding plan.	Possibility of ‘regression to mean’ (EBF rate was lower before the intervention, which increased the likelihood of a significant increase after the intervention); definition of hypothermia included 36.5°C.
5.	Kelly 2018 [9]	Maternal-newborn family-centered care unit (FCCU), USA; HIC	Non-RCT	Newborns with a gestational age of 37 wk or more born to English-speaking mothers. Exclusion criteria: gestational age less than 37 wk, hypoglycemia protocol, multiple gestation, scheduled antibiotics for the mother or the newborn, and a mother who was physically unable or unwilling to provide a minimum of 1 h of skin to skin contact after bath. n = 75 (3 h = 25, 6 h = 25, 9 h = 25). Gest = 39 wk/BW = 3.3 kg.	Three arm trial: Group 1 = bath at 9 h of age. Group 2 = bath at 6 h of age. (Mothers chose one of the three bathing time slots: 3, 6, or 9 h of age. Once the required 25 newborns per time slot were selected, mothers chose from the remaining available time slots).	Group 3 = Bath at 3 h of age (skin to skin contact provided after bath in all 3 groups).	Axillary (core) and skin temperatures before the bath; immediately after the bath; and 5, 30, 60, and 120 min after the bath.	Significant difference in axillary temperatures between the 3 h and 9 h age groups, although this difference was not clinically significant (0.1°C); no significant differences in skin temperatures among the three age groups.	Data obtained by correspondence; data for 6 h and 3 h groups clubbed together as early bath and compared with 9 h group (delayed bath); neonates with axillary temp ≤36.5°C at any time point after bath considered hypothermic.
6.	LiVolsi 2018 [17]	Community hospital, USA; HIC	Before after study	Well, non-at-risk newborns. Exclusion criteria: not mentioned. n = 419 (post-intervention = 319, pre-intervention = 100). Population characteristics not mentioned.	Bath delayed until 24 h after birth. Post-intervention period: over the two-month period following implementation in November 2017.	Bathed within 2 h of life. Pre-intervention period = not mentioned.	In-hospital breastfeeding rates, formula supplementation, hypothermia, and hypoglycaemia incidences.	Statistically non-significant improved breastfeeding rates, reduced incidence of hypoglycaemia and hypoglycaemia.	Dissertation; high risk of bias; selection of subjects not clear; outcome definitions not mentioned clearly.
7.	Long 2020 [18]	Large urban community hospital, USA; HIC	Before after study	Inclusion: mothers who expressed a desire to exclusively breastfeed on admission, had given birth to a single infant, and were admitted to the postpartum unit as a stable mother–baby couplet. Exclusion criteria: unstable mothers or infants who required a higher level of care, multiple gestation birth, or a condition that is contraindicated to breastfeed such as human immunodeficiency virus infection or illicit drug use. n = 1463 (post-intervention = 899, pre-intervention = 564). Gest = 39 wk/BW = 3-4 kg.	Delaying the first newborn bath until at least 12 h after birth. First post-delayed bath cohort: 5-plus months after the change in practice (n = 468; mean timing = 13.84 h). Second post-delayed bath cohort: additional 5 mo to evaluate a cohort for sustainability of the practice (n = 431; mean timing = 14.39 h).	First bath within 2 h of age (n = 564; mean timing 4.09 h). Pre-intervention period = 6 mo prior to the date of delayed bath implementation.	Exclusive breastfeeding during hospitalization	No significant increases in the EBF rates (baseline 74.1%) in both the first post-implementation delayed bath cohort (70.1%, *P* = 0.207) and the second “sustainability” cohort (79.4%, *P* = 0.060).	Baby-friendly designated hospital setting (high EBF rates at baseline); numbers combined from both post-intervention cohorts to compare with pre-intervention cohort.
8.	Mallick 2019 [19]	All live births at home within last 5 y in Nepal and 3 y in Bangladesh (India did not have data on bath); LMIC	Cross-sectional study	Only live births were included ie, stillbirths were excluded (using data from Demographic and Health Surveys). Cases were children who died. Excluded cases included births occurring in the 28 d preceding the survey and newborn deaths occurring on the day of birth (day 1). n = 4115 (for mortality analysis; group-wise numbers not available). Gest/BW = not mentioned.	Delayed bath: Bath after until at least 6 h after birth. (Data obtained from mothers in health surveys).	Early bath: Bath within 6 h after birth.	Neonatal mortality excluding death on day of birth (Death after day 1 of life till 28 d).	No significant difference in mortality between early bath vs delayed bath groups (OR = 1.4; 95% CI = 0.6-3.3); increased mortality in neonates with invalid maternal responses on bathing vs delayed bath (OR = 52.4, 95% CI = 18.2-150.6) but mortality could have led to the absence of a response.	Unadjusted OR provided; extremely high risk of misclassification of intervention status because of unusually strong recall bias related to outcome status (maternal responses likely affected by neonatal deaths).
9.	Mardini 2020 [8]	Maternity dept of the University Hospital Center, Lebanon; UMIC	Randomized pilot trial	Population: Newborns. Exclusion: Babies whose mothers requested to bath them earlier than 2 h. n = 125 (2 h = 51, 6 h = 51, 24 h = 23). Gest/BW = not mentioned.	Three arm trial: Group 1 = bath at 24 h of age. Delayed bath group.	Group 2 = bath at 6 h of age. Group 3 = Bath at 2 h of age. Both groups (2 and 3) included in early bath group.	Skin-to-skin contact, parent participation in bath, incubation time (time for thermal stabilization), general state of the baby.	More newborns had skin-to-skin contact with mothers when bathed at 24 h vs 2 h; more parental participation in bath at 24 h vs 6 or 2 h; more newborns calm with bath at 24 h vs 2 or 6 h; higher incubation time with bath at 2 or 6 h vs 24 h.	Axillary temp was to be recorded but not done (article still mentions reduction in hypothermia); randomization using a dice (some concerns for bias); not included in meta-analysis (data on hypothermia not available).
10.	McInerney, 2015 [5]	Community hospital, UK; HIC	Before after study	Newborns (no other information). Exclusion criteria: not mentioned. n = 1135 (post-intervention = 557, pre-intervention = 578). Population characteristics = not available.	Bath was delayed until 12 h after birth. Post-intervention period: not mentioned.	Bathed within 2 h of life. Pre-intervention period = not mentioned.	Rates of neonatal hypoglycaemia (blood glucose <49 mg/dL).	Significant decrease in incidence of hypoglycemia (3.5% vs 8.5%) in whole population as well as in “at risk” neonates (14% vs 28%).	Full text not available, data extracted from abstract; hypoglycaemia defined differently as compared to WHO.
11.	Mullany 2010 [20]	Data from large community-based RCTs of the efficacy of chlorhexidine on neonatal mortality, Nepal; LMIC	Case-control study (data from RCTs)	Babies with one or more measures of axillary temperature recorded during the home visit schedule (maximum 11 visits) were included in the analysis. Exclusion: not mentioned, n = 23 240 babies and 213 636 axillary temperatures measures (number of observations mentioned in each group rather than number of newborns). Gest, BW = not mentioned.	First bath timing classified into six categories: >24 h, 12-23.9 h, 6-11.9 h, 3-5.9 h, 1-2.9 h, <1 h.	All categories compared with the group with first bath timing >24 h.	Neonatal hypothermia: an axillary temperature of less than 35°C	No effect of timing of bath on hypothermia; other findings: In addition to season in which the babies were born, weight was an important risk factor for hypothermia. There was no effect of hat wearing, room warming or skin-to-skin contact on hypothermia.	Prevalence ratio used as effect size along with contrasting definition of hypothermia; hence could not be included in meta-analysis; timing of bathing unlikely to affect hypothermia of such severity (axillary temp <35°C).
12.	Preer 2013 [21]	Urban safety-net teaching hospital, USA; HIC	Before after study	Newborns admitted to the well infant nursery and eligible to breastfeed. Exclusion: maternal factors like HIV infection, illicit drug use, or any other contraindication to breastfeeding; mothers admitted to the intensive care unit after delivery, infants transferred to the neonatal intensive care unit, parents requested an early bath or who had their bath delayed for another reason. n = 714 (post-intervention = 366, pre-intervention = 348). <37 wk = 4%, <2500 g = 4.5% in both groups.	First bath given after 12 h of life. Parents participated in bathing the infant, and after the bath, infants were dried and placed skin-to-skin under dry blankets. (mean bath timing = 13.5 h). Post-intervention period = 6 mo after policy change	Bathed at approximately 2 h of life, or later if the neonate had yet to achieve a stable temperature; infants were dried, wrapped in dry blankets, and placed under radiant warmer (mean bath timing = 2.4 h). Pre-intervention period = 6 mo before policy change.	In-hospital exclusive breastfeeding rates, breastfeeding initiation	In-hospital EBF rates increased from 32.7% to 40.2% after the bath was delayed (AOR = 1.39; 95% CI = 1.02-1.91); increased BF initiation rates (AOR = 2.66; 95% CI = 1.29-5.46).	Co-intervention in the post-intervention cohort (skin-to-skin contact may have independently led to the measured improvement in breastfeeding rates).
13.	Shifa 2018 [22]	Gamo Gofa Zone, Southern Ethiopia; LIC	Matched case-control study	Only live births were included ie, stillbirths were excluded. Cases were children who died between March 1, 2011, and September 30, 2014, and randomly selected two live controls of under-five children born within one-month time in the same locality with each case. n = 789 infants out of total 1143 children (cases = 263, controls = 526). Gest/BW = not mentioned.	Bath after until at least 24 h after birth (n = 298 infants)	Bath within 24 h after birth (n = 491 infants)	Maternal and child health related predictors of under-five and infant mortality	Delaying first bath at least for 24 h found to be significantly associated with under-five (AOR = 0.50, 95% CI = 0.34-0.73) and infant mortality (AOR = 0.46, 95% CI = 0.28-0.77).	An extremely high risk of misclassification of intervention status because of unusually strong recall bias related to outcome status; data used for infant mortality only.
14.	Suchy 2018 [3]	Mother baby unit, USA; HIC	Before after study	Newborns with gestational ages >38 wk and no known exposure to HIV and hepatitis B in utero. Excluded: newborns requiring delivery room intervention after initial stabilizing care or where acute decompensation of the newborn's condition occurred. n = 1205 (post-intervention = 883, pre-intervention = 322). BW/gest = NA.	Delay newborn bath (immersion or tub) for 12 h and when vitals are stable. First post-delayed bath cohort: seven consecutive wk after the policy change (n = 486). Second post-delayed bath cohort: seven-week maintenance period after three months of full implementation (n = 397).	Sponge bath within first 12 h of life and when vitals are stable (n = 322). Pre-intervention period = seven wk before the change occurred.	Incidence of hypothermia (axillary temperature <36.2°C or <97.2°F) and exclusive breastfeeding rates.	Almost 100 percent of newborns had stable temperatures. Breastfeeding exclusivity rates did not change significantly: baths less than 12: 79% pre-intervention, 74% post-intervention, and 68% maintenance; baths 12 h: 68 percent pre-intervention, 71% post-intervention, and 73% maintenance.	Hypothermia definition different (<36.2°C) and data not available; co-intervention in the post-change cohort (immersion bath is reported to result in less heat loss in neonates); could not be included in meta-analysis due to distinct early bath definition (≤12 h).
15.	Turney 2019 [23]	Postpartum unit in a community hospital, USA; HIC	Before after study	Newborns born at greater than 37 wk gestational age who were admitted to the postpartum unit. Exclusion criteria: newborns born at less than 37 wk gestational age and newborns born at greater than 37 wk who were transferred to the NICU or whose mothers had HIV or hepatitis B or C. n = 1959 (post-intervention = 1343, pre-intervention = 616). Gest/BW = not mentioned.	Delayed bath for at least 9 h (mean bath timing 13.17 h). Post-intervention period = 8 m after implementation.	Bathing a newborn once his/her vital signs have stabilized (mean bath timing 6.88). Pre-implementation period = 4 m prior.	Exclusive breastfeeding rates at discharge	The rate of exclusive breastfeeding at discharge did not change significantly after implementation (*P* > 0.05), regardless of when the first bath was given.	In the phase of Baby Friendly Hospital Initiative status acquisition; intention to treat analysis for EBF rates (non-adherers included in the group as intended).
16.	Warren 2020 [24]	Provincial children’s hospital, Canada; HIC	Before after study	Newborns born during the specified time periods who were 34 wk gestation or older and whose mothers were admitted to the maternity unit. Exclusion criteria: Newborns for whom delaying the bath or breastfeeding was contraindicated; newborns of women using illicit drugs or on methadone treatment; and newborns born to women with HIV, hepatitis B, hepatitis C, active HSV infection, and Methicillin Resistant Staph aureus; newborns whose mothers were admitted to ICU, newborns admitted to the NICU from the birthing room, and newborns not bathed in the time frame directed by the policy (ie, born before the policy change but bathed after 24 h and born after the policy change but bathed before 24 h). n = 1225 (post-intervention = 545, pre-intervention = 680). Gest = 39 wk/BW = not mentioned.	Delaying the first newborn bath until at least 24 h after birth (Mean timing of bath = 30 h); after bathing, mothers were encouraged to place their newborns skin to skin, or newborns were wrapped and given to their mothers. Post-implementation period: from the 6-mo period of July – December 2015	Newborns from birthing room transported to the nursery, bathed in a tub, and then wrapped them in warmed blankets or placed them under a radiant warmer before returning them to their mothers (Mean timing of bath = 3.5 h). Pre-implementation period: from the 6-mo period of June through November 2014	Exclusive breastfeeding rates, breastfeeding initiation rates, hypothermia and hypoglycaemia incidences.	Higher odds of EBF at discharge in the post-change group than in the pre-change group (AOR = 1.334; 95% CI = 1.05-1.70; *P* = 0.019); decreased incidence of hypothermia (*P* = 0.007) and hypoglycemia (*P* = 0.003); no difference in breastfeeding initiation between groups.	Co-intervention in the post-intervention cohort (skin-to-skin contact after birth and/or after bath may have led to the observed improvement in the outcomes); increased awareness of Baby-Friendly practices may have altered the EBF rate as well.

HIC – high-income country, LIC – low-income country, LMIC – lower middle-income country, RCT – randomized controlled trial, UMIC – upper middle-income country, wk – week, h – hour, mo – month, BW – birth weight, gest – gestation, kg – kilograms, NICU – neonatal intensive care unit, OR – odds ratio, AOR – adjusted odds ratio, CI – confidence interval, mg/dL – milligram per decilitre, EBF – exclusive breastfeeding

Due to variation across studies in the definitions of early and delayed bath, we performed the analysis as follows: a pre-specified analysis was done for studies which defined early and delayed bath as in our review protocol, ie, delayed bath after 24 h of age compared to early bath before 24 h of age (ie, first bath at >24 h vs ≤24 h of age); and a post-hoc analysis for the most commonly used timing of the first bath in included studies, ie, delayed bath after 6 h of age compared to early bath before 6 h of age (ie, first bath at >6 h vs ≤6 h of age).

### Design

The designs of the included studies were before-after (n = 11), case-control (n = 2), randomized trial (n = 1), non-randomized trial (n = 1) and cross-sectional (n = 1). Before-after studies compared neonatal outcomes pre- and post-implementation of a new policy for delaying first bath in the hospital. Mullany et al. [20] performed a case-control study based on data from large community-based RCTs in Nepal, looking at the association of risk factors (including timing of the first bath) and hypothermia. Another case-control study was conducted in Ethiopia to look at the maternal and child health-related predictors of under-five and infant mortality [22]. A 3-arm randomized trial studied the effects of 2-, 6- and 24-hour baths on newborn stabilization and behaviour [8]. In another 3-arm but non-randomized trial, the authors compared the effects of 3-, 6- and 9-hour baths on axillary temperature of the newborns [9]. Using data from the Demographic and Health Surveys, Mallick et al [19] studied neonatal thermal care and umbilical cord care practices in South Asia along with their associated mortality data.

### Setting

The included studies were conducted in high-income (Canada, UK, and USA, n = 12), upper middle-income (Lebanon; n = 1), lower middle-income (Nepal and Pakistan, n = 2) and low-income (Ethiopia, n = 1) countries. The facility-based studies (11 before-after studies and 2 trials) enrolled neonates admitted in the community hospitals or mother-baby units. Three studies were based on data from the community in low- and middle-income countries (LMICs).

### Participants

The review included 16 studies involving 39 020 neonates. 12 studies involving 14 421 neonates contributed to the meta-analysis. The participants were term and near-term healthy neonates, without any major comorbidities (sickness, ineligibility for breastfeeding, anomalies, etc.). Population characteristics were not clearly mentioned in six studies [5,14,17,19,20,22]; however, there was no indication to suggest that the newborns were not healthy.

### Intervention

#### Details of intervention

In facility-based studies, the timing of first bath was noted either prospectively or collected retrospectively from patient records in the hospital. These details in case-control studies were available from survey data collected in interviews from the caretakers conducted after a significant time interval from the event (first bath).

#### Timing of delayed bathing

Delayed bathing was defined variably by the included studies (Table S1 in **Online Supplementary Document**). Six studies defined delayed bathing as first bath at least 24 h after birth [8,13,15,17,22,24]. Among these, the mean bathing timing was mentioned as 30 h in one study [24].

Five studies defined delayed bath as first bath at least 12 h after birth. The mean timing of first bath was 17.9 [16], 14 [18], and 13.5 h [21] in three studies and not specified in two studies [3,5]. The cut-off of 9 h was used for delayed bathing by one study (mean time 13 h) [23]. In three-arm trials, one study compared 3-, 6- and 9-hour baths [9], while another study compared 2-, 6- and 24-hour baths [8]. In these trials, we considered 9- and 24-hour baths as delayed, respectively, and baths at ≤6 h (2-, 3- or 6-hour baths) as “early” to uniformly align the definition of early bath (≤6 h) in meta-analysis. One study classified first bath timing into six categories: >24, 12-23.9, 6-11.9, 3-5.9, 1-2.9 and <1 hour [20]. All groups were compared against “>24-hour group” for the outcome of hypothermia in this study. One study did not define the exact timing of baths but mentioned mean timings (16 h for delayed and 4.6 h for early bath) [14].

#### Adherence to intervention

Two studies mentioned the number of neonates who did not adhere to the intervention and were bathed earlier [3,23]. There was no mention of the adherence rate to delayed bathing policy in other studies.

#### Cointervention

Two studies included skin-to-skin contact after bath in the new delayed bathing policy [21,24]. Two studies introduced a policy change which involved delaying the first bath as well as changing the type of bath (immersion bath instead of sponge bath) [3,13]. Three studies mentioned the possibility of potential impact of baby friendly hospital initiative (BFHI) program on breastfeeding rates [18,23,24].

### Comparison

The timing of “early” bath also varied across the studies, with different cut-off definitions (Table S1 in **Online Supplementary Document**). The most common definition of early first bath was bath within the first 6 h of life (12 studies). In one study, first bath within 12 h after birth was considered as early bath [3]. Neonates were bathed within 2-4 h after birth (early bath) in five studies, which was a routine care practice in the pre-implementation phase [5,13,17,18,21]. Some studies reported the mean timing of early bath rather than a cut-off as 1.9 h [16], 6.9 h [23] and 3.5 h [24]. One study used six different time-periods for comparison [20]. In two studies, we defined first bath at or within 6 h of life, ie, at 2, 3 or 6 h after birth as early [8,9].

In two studies, 25%-27% of the newborns did not adhere to the timing of early bathing (control) in pre-implementation phase and received their first baths later [3,23]. There was insufficient information in the rest of the studies to clearly rule out contamination in the pre-implementation (control) group.

### Outcomes

The critical outcomes reported by the included studies were neonatal and infant mortality, hypothermia, hypoglycemia, and exclusive breastfeeding (EBF) rates at discharge. None of the included studies reported sepsis or possible serious bacterial infection, or exclusive breastfeeding rates at 6 months.

Neonatal and infant mortality was reported by single studies [19,22]. One study reported the effect of delayed bath on neonatal mortality by analysing the health survey data from 4115 participants in Bangladesh and Nepal [19]. The authors included neonatal deaths after day one of life till 28 days (excluding deaths on day of birth because these were unlikely to be related to bath practices).

Hypothermia and hypoglycaemia were reported by eight and four studies, respectively, six and three of which could be included in meta-analysis, respectively. A neonate who had at least one episode of hypothermia or hypoglycaemia after bath during hospital stay was considered to have an event. One study defined hypoglycaemia as blood glucose <49 mg/dL [5]; however, we pooled the study results with other studies (which used WHO definition of blood glucose <45 mg/dL) due to similarity in cut-off values.

Eight studies reported EBF rates at discharge, seven of which could be included in meta-analysis. Most studies used same definition for EBF (having received only breastmilk and no formula, water, or glucose water during the birth hospitalization); however, some studies did not provide a clear definition of EBF [15-17].

Three studies reported breastfeeding initiation, defined as any act of putting the baby to mother’s breast [16,21,24].

### Risk of bias in included studies

A summary of the risk of bias assessment is provided in Appendix S3 in **Online Supplementary Document**. Six studies were judged to be at critical risk, and 10 studies at serious risk of bias mostly due to confounding effect.

### Effects of interventions

The results are summarized separately for pre-specified analysis (first bath at >24 h vs ≤24 h of age) and post-hoc analysis (first bath at >6 h vs ≤6 h of age) (**Table 2**).

**Table 2:** Summary of findings: Delayed bath vs early bath in term healthy newborns

Outcomes	No. of participants (studies) follow-up	Certainty of the evidence (GRADE)	Relative effect (95% CI)	Anticipated absolute effects	
**Risk with early bath**	**Risk difference with delayed bath**¶
Infant mortality (Delayed bath (>24 h) vs Early bath (≤24 h))	789 (1 observational study)	Low*	OR = 0.46 (0.28-0.77)	397 per 1000	165 fewer per 1000 (241 fewer to 61 fewer)
Hypothermia (Delayed bath (>24 h) vs Early bath (≤24 h))	660 (1 observational study)	Low*	OR = 0.50 (0.28-0.88)	130 per 1000	61 fewer per 1000 (90 fewer to 14 fewer)
Exclusive breastfeeding at discharge (Delayed bath (>24 h) vs Early bath (≤24 h))	660 (1 observational study)	Very low*†	OR = 0.81 (0.58-1.12)	621 per 1000	51 fewer per 1000 (134 fewer to 26 more)
Neonatal mortality (Delayed bath (>6 h) vs Early bath (≤6 h))	(1 observational study)	Very low*†	OR = 0.71 (0.30-1.67)	Not available	Not available
Hypothermia (Delayed bath (>6 h, ie, at or after 9, 12 or 24 h) vs Early bath (≤6 h))	3582 (5 observational studies)	Low‡§	OR = 0.23 (0.10-0.54)	147 per 1000	109 fewer per 1000 (130 fewer to 62 fewer)
Hypoglycaemia (Delayed bath (>6 h, ie, at or after 12 or 24 h) vs Early bath (≤6 h))	2775 (3 observational studies)	Low*	OR = 0.39 (0.23-0.66)	49 per 1000	30 fewer per 1000 (38 fewer to 16 fewer)
Exclusive breastfeeding at discharge (Delayed bath (>6 h, ie, at or after 9, 12 or 24 h) vs Early bath (≤6 h))	6768 (6 observational studies)	Moderate‡	OR = 1.20 (1.08-1.34)	584 per 1000	44 more per 1000 (19 more to 69 more)

CI – confidence interval, OR – odds ratio, h – hour

*Most of the pooled effect provided by studies at “critical risk of bias”.

†Wide confidence interval crossing the line of no effect.

‡Most of the pooled effect provided by studies at “serious risk of bias”.

§Significant heterogeneity (*I^2^* statistics ≥60%).

¶The risk in the intervention group (and its 95% CI) is based on the assumed risk in the comparison group and the relative effect of the intervention (and its 95% CI).

#### Pre-specified analysis: Delayed first bath (>24 h after birth) vs early bath (≤24h after birth)

Two studies used the protocol-defined cut-off of delayed bath (>24 hours) vs early bath (≤24h) [15,22].

Infant mortality was reported by one study [22]. 23% of infants died in the delayed bathing (>24h) compared to 40% infant deaths in the early bathing group (≤24h) (adjusted OR = 0.46, 95% CI = 0.28-0.77; low certainty evidence).

Hypothermia during hospital stay was reported by one study [15]. 7% of the newborns developed hypothermia in the delayed bathing (>24h) compared to 13% newborns in the early bathing group (≤24h) (OR = 0.50; 95% CI = 0.28-0.88; low certainty evidence).

EBF rate at discharge was reported by one study [15]. 57% of newborns were breastfeeding exclusively at discharge in the delayed compared to 62% the early bathing group (OR = 0.81, 95% CI = 0.58-1.12; very low certainty evidence).

#### Post-hoc analysis: Delayed first bath (>6 h, ie, at or after 9, 12 or 24 h after birth) vs early bath (≤6 after birth)

We could pool the results of ten studies under this comparison because the mean timings of early first bath in all these studies were within the first six h of life. The mean timing of early bath was 6.9 h in one study but was included in meta-analysis due to proximity to the cut-off timing (6 h) [23].

Neonatal mortality was reported by one study [19]. The unadjusted OR was 0.71 (95% CI = 0.30-1.67; very low certainty evidence) for delayed first bath (>6 h after birth) compared to early bath (within 6 h after birth).

**Figure 2:**
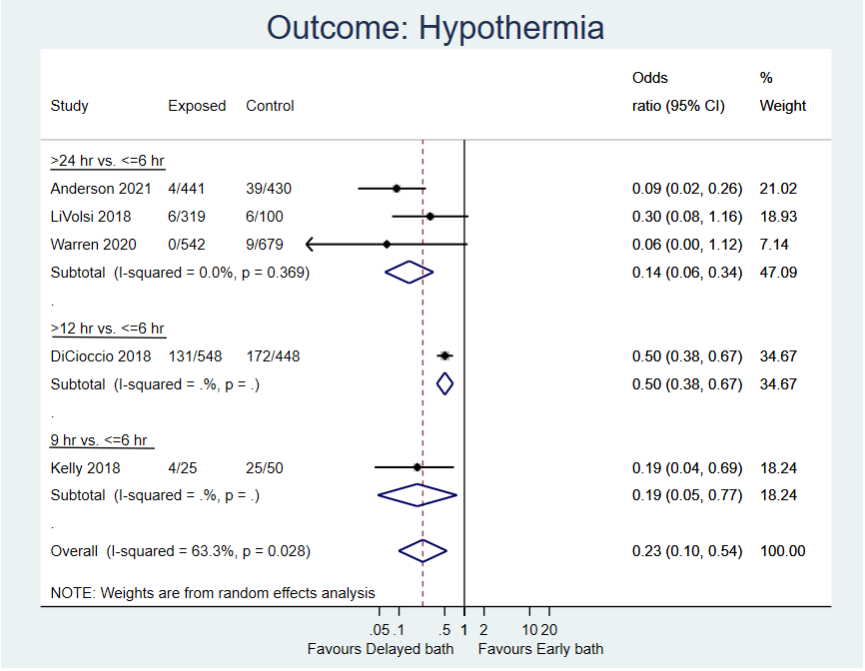
Forest plot for post-hoc analysis: Delayed first bath (>6 hours, ie, at or after 9, 12 or 24 hours after birth) vs early bath (≤6 hours after birth) in term, healthy newborns. Outcome: Incidence of hypothermia.

Ten percent of the newborns, who bathed more than 6 h after birth, developed hypothermia compared to 17% of those bathed within 6 h after birth (OR = 0.23, 95% CI = 0.10-0.54; five studies, 3582 newborns; low certainty evidence; **Figure 2**).

**Figure 3:**
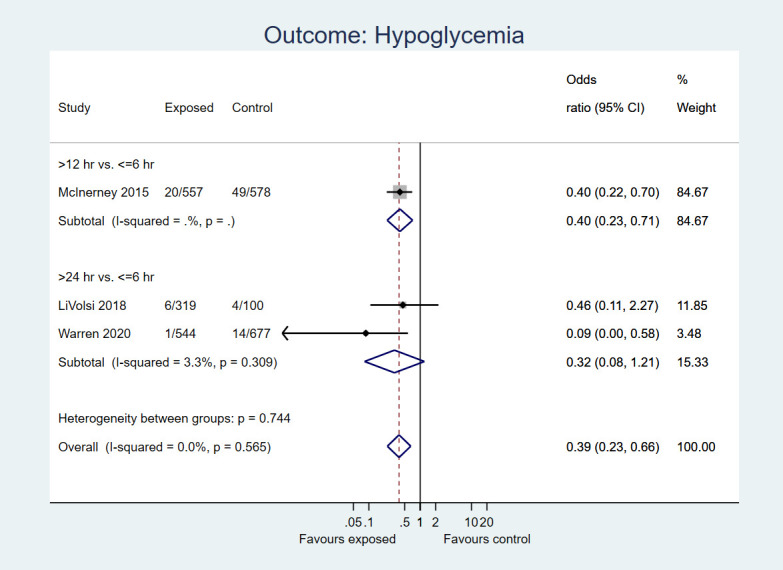
Forest plot for post-hoc analysis: Delayed first bath (>6 hours, ie, at or after 12 or 24 hours after birth) vs early bath (≤6 hours after birth) in term, healthy newborns. Outcome: Incidence of hypoglycaemia.

Three studies including data on 2775 neonates reported hypoglycaemia. There was 61% decrease in the odds of hypoglycaemia in neonates who underwent delayed bath (>6 h) compared to early bath (≤6 h) (OR = 0.39, 95%CI = 0.23-0.66; low certainty evidence; **Figure 3**).

**Figure 4:**
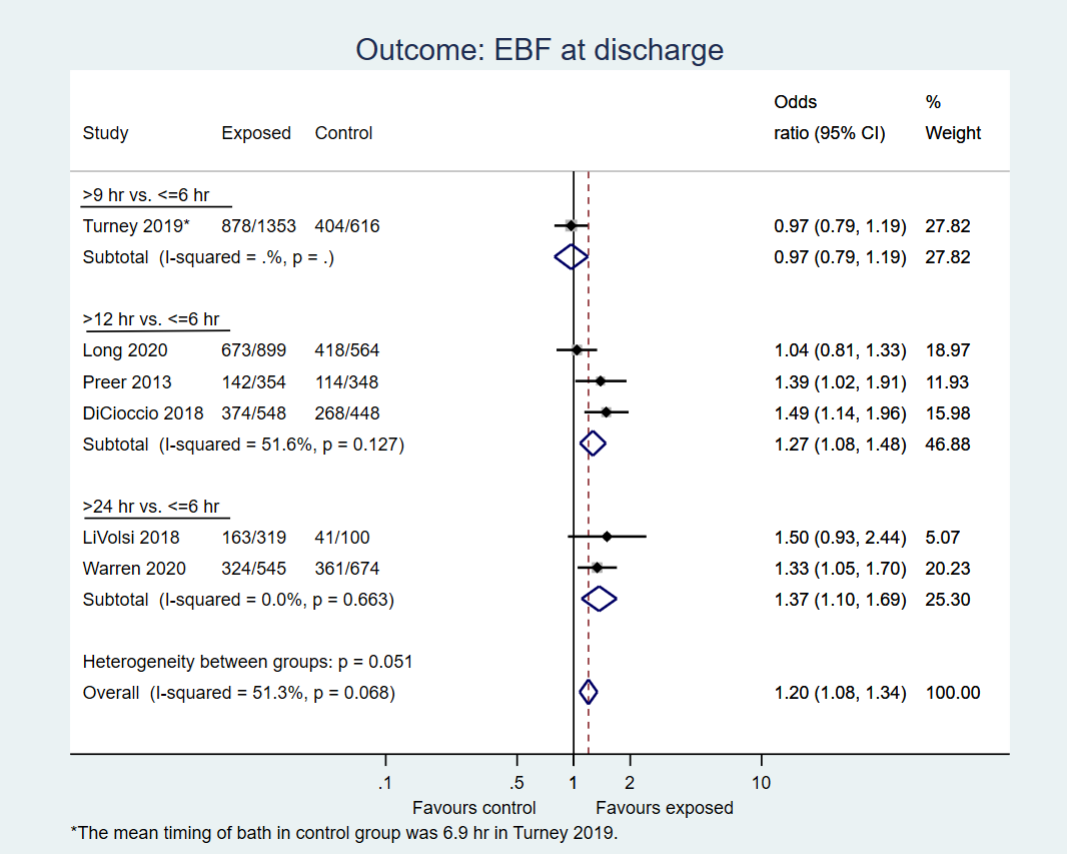
Forest plot for post-hoc analysis: Delayed first bath (>6 hours, ie, at or after 9, 12 or 24 hours after birth) vs early bath (≤6 hours after birth) in term, healthy newborns. Outcome: Incidence of exclusive breastfeeding rates at hospital discharge.

Six studies including 6768 neonates reported EBF at discharge. The pooled OR was 1.20, favouring delayed bath (95% CI = 1.08-1.34; moderate certainty evidence; **Figure 4**).

Three studies reported proportion of neonates initiated on breastfeeding post-implementation of a delayed bath policy in 2221 neonates. There was no difference in the odds of breastfeeding initiation based on timing of the bath (OR = 1.35; 95% CI = 0.86-2.13; Figure S3 in **Online Supplementary Document**).

## DISCUSSION

The results of the current review suggest that delaying first newborn bath for at least 24 h after birth may reduce infant mortality and neonatal hypothermia compared to early bath within first 24 h. The effect of delaying bathing for at least 24 h on EBF at discharge is very uncertain. Delaying bathing for at least 6 h after birth probably improves EBF at discharge and may reduce the risk of hypothermia and hypothermia during hospital stay. The effect of delayed bath for at least 6 h on neonatal mortality is very uncertain.

No prior reviews have evaluated the effect of delaying the first newborn bath on neonatal outcomes. Our findings in the review support the existing WHO recommendations to delay newborn bathing for at least 24 h after birth and if not possible, for at least 6 h after birth [7]. This recommendation, however, was based on expert consensus, but the evidence generated by this review is supportive of the same.

The scarcity of evidence on newborn bath practices was apparent in a recent survey on newborn skincare policies across United States maternity hospitals [25]. 87% of the surveyed 109 US hospitals practised delaying the first newborn bath by at least 6 h, but the evidence for these policies cited by hospitals was unclear. This was also evident in our review, as there were no large randomized trials evaluating the effects of delaying first bath systematically.

The findings in the review are supported by plausible biological mechanisms. Mardini et al [8] showed that delayed bath beyond 24 h was associated with vernix caseosa retention on the skin and adequate time for skin-to-skin contact with mother. Vernix caseosa may act an important role in preventing evaporative water loss, thermoregulation, and innate immunity [26]. This, along with better STS contact opportunities, can explain the lesser incidence of hypothermia and hypoglycaemia and better breastfeeding rates.

This review tried to answer an important research question on the effect of delayed first newborn bath on mortality and morbidities in term healthy neonates. Rigorous methodology was followed to conduct this review, with an all-inclusive literature search and no language filters. Though this review included 16 studies, there were no RCTs in the meta-analysis. One pilot RCT involved a small number of neonates and did not contribute to the meta-analysis [8]. Moreover, there is no uniformity in definition of delayed and early baths in literature. For example, two studies were excluded due to comparison of baths over a narrow time period (1- and 4-hour baths) or too wide a time period (24- and 48-hour baths) [27,28]. Thus, there is a scarcity of good quality trials that have assessed the impact of delayed first bath on important health outcomes in term healthy newborns. There is also a paucity of literature on the process of bathing (type of bath, water temperature, environment temperature, bathing products, any risk of adverse events like slippage or drowning, need for counselling etc.), which remains to be addressed by research.

None of the included studies reported outcomes of any serious morbidities (sepsis or possible serious bacterial infection), timing of breastfeeding initiation, exclusive breastfeeding rates at 6 months or any adverse events related to intervention. There was a wide variation in the definition of delayed and early first bath in the included studies. This led to difficulty in comparison of interventions across the studies. However, we could meta-analyse ten studies together after realizing the timing in early bath group to be within the first 6 h of life in all these studies. The evidence for comparison of delayed bath >24 h vs early bath <24 h was based on single study results. The evidence was rated as low- and very low-quality, in part, affected by very serious risk of bias. A few late preterm neonates (two trials, 1646 newborns) without major comorbidities were among the studied population. We did not consider this to be serious indirectness because they were otherwise healthy newborns which did not downgrade the evidence. Certain cointerventions could have independently affected the outcomes in the studies. For example, immersion (tub) bath is known to decrease heat loss during bath, and hence, it could have affected the temperatures of the neonates independently.

## CONCLUSIONS

Delaying the first newborn bath for at least 24 h after birth may reduce infant mortality and hypothermia. Additionally, delayed first bath for at least 6 h after birth may prevent hypothermia and hypoglycemia, and likely improves EBF rates at discharge in healthy newborns. The available evidence supports delaying the first newborn bath by 24 h and, if not possible, by at least six h to improve thermoregulation and breastfeeding rates in term healthy newborns. However, most of these conclusions are based on low certainty evidence and needs further evaluation in well-designed randomized trials.

## Additional material


Online Supplementary Document

## References

[1] Dyer JA Newborn skin care. Semin Perinatol. 2013;37:3-7. 10.1053/j.semperi.2012.11.008 23419756

[2] Taşdemir Hİ Efe E The effect of tub bathing and sponge bathing on neonatal comfort and physiological parameters in late preterm infants: A randomized controlled trial. Int J Nurs Stud. 2019;99:103377. 10.1016/j.ijnurstu.2019.06.008 31442786

[3] Suchy C Morton C Ramos RR Ehrgott A Quental MM Burridge A Does Changing Newborn Bath Procedure Alter Newborn Temperatures and Exclusive Breastfeeding? Neonatal Netw. 2018;37:4-10. 10.1891/0730-0832.37.1.4 29436352

[4] Ruschel LM Pedrini DB da Cunha MLC Hypothermia and the newborn’s bath in the first hours of life. Rev Gaúcha Enferm. 2018;39:e20170263. 30365754 10.1590/1983-1447.2018.20170263

[5] McInerney CM Gupta A Delaying the first bath decreases the incidence of neonatal hypoglycemia. J Obstet Gynecol Neonatal Nurs. 2015;44:S73-4. 10.1111/1552-6909.12650

[6] Moore ER Bergman N Anderson GC Medley N Early skin-to-skin contact for mothers and their healthy newborn infants. Cochrane Database Syst Rev. 2016;11:CD003519. 10.1002/14651858.CD003519.pub4 27885658 PMC6464366

[7] WHO. WHO recommendations on postnatal care of the mother and newborn. World Health Organization; 2014. Available: https://apps.who.int/iris/handle/10665/97603. Accessed: 4 Dec 2020. 24624481

[8] Mardini J Rahme C Matar O Abou Khalil S Hallit S Fadous Khalife M-C Newborn’s first bath: any preferred timing? A pilot study from Lebanon. BMC Res Notes. 2020;13:430. 10.1186/s13104-020-05282-0 32928289 PMC7491191

[9] Kelly PA Classen KA Crandall CG Crenshaw JT Schaefer SA Wade DA Effect of Timing of the First Bath on a Healthy Newborn’s Temperature. J Obstet Gynecol Neonatal Nurs. 2018;47:608-19. 10.1016/j.jogn.2018.07.004 30096281

[10] Sterne JAC Savović J Page MJ Elbers RG Blencowe NS Boutron I RoB 2: a revised tool for assessing risk of bias in randomised trials. BMJ. 2019;366:l4898. 10.1136/bmj.l4898 31462531

[11] Sterne JA Hernán MA Reeves BC Savović J Berkman ND Viswanathan M ROBINS-I: a tool for assessing risk of bias in non-randomised studies of interventions. BMJ. 2016;355:i4919. 10.1136/bmj.i4919 27733354 PMC5062054

[12] GRADEpro GDT: GRADEpro Guideline Development Tool [Software]. McMaster University, 2020 (developed by Evidence Prime, Inc.). Available: https://gradepro.org. Accessed: 1 July 2022.

[13] Anderson J An Organization-Wide Initiative to Implement Parent-Performed, Delayed Immersion Bathing. Nurs Womens Health. 2021;25:63-70. 10.1016/j.nwh.2020.11.006 33450241

[14] Brennan RA Obrist M Olson K Implementation of Newborn Delayed-Immersion Swaddle Bathing in a Mother–Baby Unit. J Obstet Gynecol Neonatal Nurs. 2020;49:S75. 10.1016/j.jogn.2020.09.131

[15] Chamberlain J McCarty S Sorce J Leesman B Schmidt S Meyrick E Impact on delayed newborn bathing on exclusive breastfeeding rates, glucose and temperature stability, and weight loss. J Neonatal Nurs. 2019;25:74-7. 10.1016/j.jnn.2018.11.001

[16] DiCioccio HC Ady C Bena JF Albert NM Initiative to Improve Exclusive Breastfeeding by Delaying the Newborn Bath. J Obstet Gynecol Neonatal Nurs. 2019;48:189-96. 10.1016/j.jogn.2018.12.008 30677407

[17] LiVolsi K. Improving Neonatal Outcomes Through the Implementation of a Delayed Bathing Program. Seton Hall Univ DNP Final Proj. 2018. Available: https://scholarship.shu.edu/final-projects/26. Accessed: 1 July 2022.

[18] Long K Rondinelli J Yim A Cariou C Valdez R Delaying the First Newborn Bath and Exclusive Breastfeeding. MCN Am J Matern Child Nurs. 2020;45:110-5. 10.1097/NMC.0000000000000606 32097223

[19] Mallick L Yourkavitch J Allen C Trends, determinants, and newborn mortality related to thermal care and umbilical cord care practices in South Asia. BMC Pediatr. 2019;19:248. 10.1186/s12887-019-1616-2 31331315 PMC6647093

[20] Mullany LC Katz J Khatry SK LeClerq SC Darmstadt GL Tielsch JM Neonatal hypothermia and associated risk factors among newborns of southern Nepal. BMC Med. 2010;8:43. 10.1186/1741-7015-8-43 20615216 PMC2911389

[21] Preer G Pisegna JM Cook JT Henri A-M Philipp BL Delaying the bath and in-hospital breastfeeding rates. Breastfeed Med. 2013;8:485-90. 10.1089/bfm.2012.0158 23635002

[22] Shifa GT Ahmed AA Yalew AW Maternal and child characteristics and health practices affecting under-five mortality: A matched case control study in Gamo Gofa Zone, Southern Ethiopia. PLOS ONE. 2018;13:e0202124. 10.1371/journal.pone.0202124 30110369 PMC6093655

[23] Turney J Lowther A Pyka J Mollon D Fields W Delayed Newborn First Bath and Exclusive Breastfeeding Rates. Nurs Womens Health. 2019;23:31-7. 10.1016/j.nwh.2018.12.003 30593766

[24] Warren S Midodzi WK Allwood Newhook L-A Murphy P Twells L Effects of Delayed Newborn Bathing on Breastfeeding, Hypothermia, and Hypoglycemia. J Obstet Gynecol Neonatal Nurs. 2020;49:181-9. 10.1016/j.jogn.2019.12.004 32057686

[25] Wisniewski JA Phillipi CA Goyal N Smith A Hoyt AEW King E Variation in Newborn Skincare Policies Across United States Maternity Hospitals. Hosp Pediatr. 2021;11:1010-9. 10.1542/hpeds.2021-005948 34462323

[26] Singh G Archana G Unraveling the mystery of vernix caseosa. Indian J Dermatol. 2008;53:54-60. 10.4103/0019-5154.41645 19881987 PMC2763724

[27] Gözen D Çaka SY Beşirik SA Perk Y First bathing time of newborn infants after birth: A comparative analysis. J Spec Pediatr Nurs. 2019;24:e12239. 10.1111/jspn.12239 30887671

[28] Behring A Vezeau T Fink R Timing of the Newborn First Bath: A Replication. Neonatal Netw. 2003;22:39-46. 10.1891/0730-0832.22.1.39 12597090

