# Supplementary material for: Timing of first bath in term healthy newborns: A systematic review
Source: J Glob Health. 2022 Aug 17;12:12004. doi: 10.7189/jogh.12.12004 (PMC9380966; doi:10.7189/jogh.12.12004)
Supplement: Online Supplementary Document [file jogh-12-12004-s001.pdf]

ONLINE SUPPLEMENTARY DOCUMENT

Title: Timing of First Bath in Term Healthy Newborns: A Systematic Review **Authors:** Mayank Priyadarshi, Bharathi Balachander, Shuchita Gupta, Mari Jeeva Sankar

Appendix S1. Search Strategy

We used the following search terms for MEDLINE: (Newborn OR infant OR neonat*) AND (bath* OR sponging OR wash). Similar terms were used for searching the following databases: Cochrane Central Register of Controlled Trials, EMBASE and CINAHL.

Appendix S2. Results from studies with qualitative data

Four studies could not be included in meta-analysis either due to non-availability of data (number of participants not mentioned in Brennan 2020; incidence of hypothermia not reported in Mardini 2020), use of a different effect estimate (prevalence ratio in Mullany 2010) or a distinct cut-off for the control group (early bath <12 hours in Suchy 2018).

In Brennan 2020, the authors did not mention number of participants in their study, but reported a significant increase in the mean temperature of newborns after bath, from 98.2 F (36.7 C) to 98.5 F (36.9 C), post-implementation of delayed bath policy. No significant difference, however, was noted on breastfeeding rates at discharge. In the 3-arm trial by Mardini 2020, newborns bathed at 24 hours were shown to have lower thermal stabilization time, more skin-to-skin contact with mothers, reduced crying and more parental participation, compared to early bathing at 2 and 6 hours.

The effect of different timings of first bath on hypothermia was presented as prevalence ratios in Mullany 2010, with a different definition for hypothermia (<35.0^{o} C). Based on 182,573 observations, the adjusted prevalence rate ratios (95% CI), as compared with delayed bath >24 hours were 0.97 (0.56 to 1.71), 0.98 (0.57 to 1.67), 1.21 (0.69 to 2.12), 1.05 (0.53 to 1.51) and 0.90 (0.53 to 1.51) for baths given at 12-23.9, 6-11.9, 3-5.9, 1-2.9 and <1 hour, respectively. These results did not suggest any significant effect of bath timing on hypothermia.

Suchy 2018, including 1205 neonates, did not report any alteration of hypothermia incidence (axillary temperature <36.2 C) with the introduction of a delayed bathing program (>12 hours). The results also did not show any significant effect of delayed immersion bath policy on EBF rates at discharge (OR 0.80; 95% CI 0.59 to 1.08).

Chamberlain 2018 found a statistically significant decrease in the number of hypoglycemia episodes (blood glucose ≤45 mg/dL) post-intervention (330 neonates; delaying bath >24 hours) compared to preintervention (330 neonates; bath ≤24 hours). However, they presented this data in non-interpretable form making it difficult to include in meta-analysis.

Appendix S3. Risk of Bias assessment

A summary of the risk of bias assessment in the included studies is depicted in Figure S1 and Figure S2. 12 studies were at serious risk of bias for confounding because these studies either did not address the potential confounders (like birth weight, intrauterine growth status and gestational age) or did not adjust for these confounders. Two studies (LiVolsi 2018 and McInerny 2015) did not specify any selection

criteria for the study participants. Three studies (case-control and cross-sectional design) were at critical/ serious risk of bias for misclassification of interventions due to recall bias. Three studies were at critical/ serious risk of bias for deviation from intended interventions. This was due to co-intervention in Preer 2013 (STS contact with mother allowed with delayed bath) and contamination in Long 2020 and Turney 2019 (poor adherence rate to intervention). The only randomized trial (Mardini 2020) was at serious risk of bias due to randomization process (rolling a dice) and little to no information for most other domains.

Table S1. Timing of first bath in included studies

No. of studies	Delayed bath	Early bath	Study (sample size)
2	>24 h	≤24 h	Chamberlain (660)<br>Shifa (789)
4	>24 h	≤6 h	Anderson (900) Livolsi (419)<br>Mardini* (125)<br>Warren (1225)
5	>12 h	≤6 h	Brennan* (NA)<br>DiCoccio (996)<br>Long (1463)<br>McInerney (1135)<br>Preer (714)
2	≥9 h	≤6 h	Kelly (75) Turney (1959)
1	>6 h	≤6 h	Mallick (4115)
1	>12 h	≤12 h	Suchy* (1205)
1	>24 h	Different timings	Mullany* (23240)

*Not included in meta-analysis

Table S2: Grade evidence profile

Certainty assessment							№ of patients		Effect		Certainty	Importance
№ of studies	Study design	Risk of bias	Inconsistency	Indirectness	Imprecision	Other considerations	delayed bath	early bath (<=6 hours)	Relative (95% CI)	Absolute (95% CI)		

Infant mortality [Delayed bath (>24 hours) vs Early bath (≤24 hours)]

1	observational studies	very serious [a]	not serious	not serious	not serious	none	68/298 (22.8%)	195/491 (39.7%)	**OR 0.46** (0.28 to 0.77)	**165 fewer per 1,000** (from 241 fewer to 61 fewer)	⊕⊕◯◯ Low	CRITICAL

Hypothermia [Delayed bath (>24 hours) vs Early bath (≤24 hours)]

1	randomised trials	very serious [a]	not serious	not serious	not serious	none	23/330 (7.0%)	43/330 (13.0%)	**OR 0.50** (0.28 to 0.88)	**61 fewer per 1,000** (from 90 fewer to 14 fewer)	⊕⊕◯◯ Low	CRITICAL

Exclusive breastfeeding at discharge [Delayed bath (>24 hours) vs Early bath (≤24 hours)]

1	observational studies	very serious [a]	not serious	not serious	serious [b]	none	188/330 (57.0%)	205/330 (62.1%)	**OR 0.81** (0.58 to 1.12)	**51 fewer per 1,000** (from 134 fewer to 26 more)	⊕◯◯◯ Very low	CRITICAL

Neonatal mortality [Delayed bath (>6 hours) vs Early bath (≤6 hours)]

1	observational studies	very serious [a]	not serious	not serious	serious [b]	none	Not available	Not available	**OR 0.71** (0.30 to 1.67)	**1 fewer per 1,000** (from 2 fewer to 0 fewer)	⊕◯◯◯ Very low	CRITICAL

Hypothermia [Delayed bath (>6 hours, i.e, at or after 9, 12 or 24 hr) vs Early bath (≤6 hours)]

5	observational studies	serious [c]	serious [d]	not serious	not serious	none	145/1875 (7.7%)	251/1707 (14.7%)	**OR 0.23** (0.10 to 0.54)	**109 fewer per 1,000** (from 130 fewer to 62 fewer)	⊕⊕◯◯ Low	CRITICAL

Hypoglycemia [Delayed bath (>6 hours, i.e, at or after 12 or 24 hr) vs Early bath (≤6 hours)]

Certainty assessment	№ of patients	Effect	Certainty	Importance

№ of studies	Study design	Risk of bias	Inconsistency	Indirectness	Imprecision	Other considerations	delayed bath	early bath (<=6 hours)	Relative (95% CI)	Absolute (95% CI)		
3	observational studies	very serious [a]	not serious	not serious	not serious	none	27/1420 (1.9%)	67/1355 (4.9%)	**OR 0.39** (0.23 to 0.66)	**30 fewer per 1,000** (from 38 fewer to 16 fewer)	⨁⨁◯◯ Low	CRITICAL
**Exclusive breastfeeding at discharge [Delayed bath (>6 hours, i.e, at or after 9, 12 or 24 hr) vs Early bath (≤6 hours)]**												
6	observational studies	serious [c]	not serious	not serious	not serious	none	2554/4018 (63.6%)	1606/2750 (58.4%)	**OR 1.20** (1.08 to 1.34)	**44 more per 1,000** (from 19 more to 69 more)	⨁⨁⨁◯ Moderate	CRITICAL

CI: confidence interval; **OR:** odds ratio

Explanations

a. Most of the pooled effect provided by studies at "critical risk of bias".
b. Wide confidence interval crossing the line of no effect.
c. Most of the pooled effect provided by studies at "serious risk of bias"
d. Significant heterogeneity (I2 stat ≥ 60%)

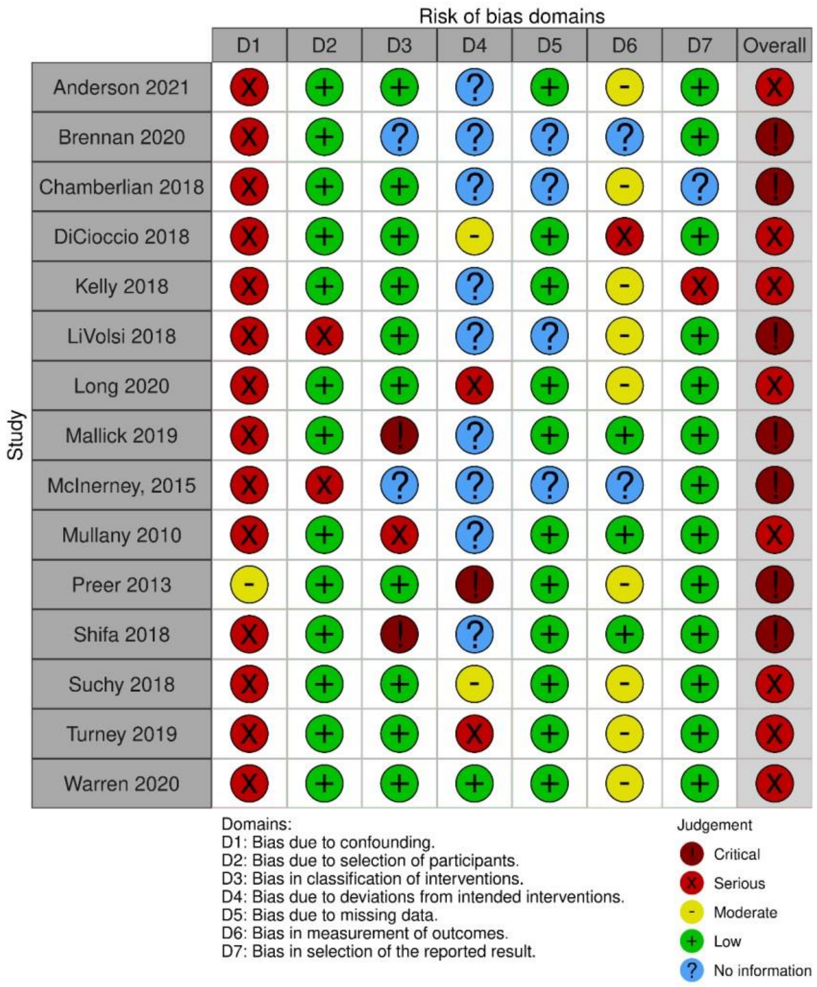

Figure S1. Risk of bias “traffic light” plots: review authors' judgements about each risk of bias item for each included study (Mardini 2020 was a randomized trial and assessed with ROB 2.0 tool, hence not shown here)

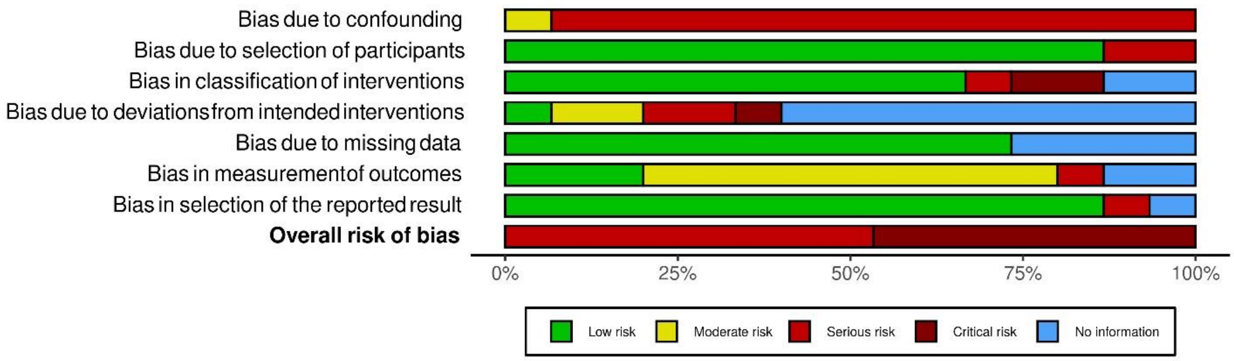

Figure S2. Risk of bias "weighted bar plots": review authors' judgements about each risk of bias item presented as percentages across the included studies (excluding Mardini 2020)

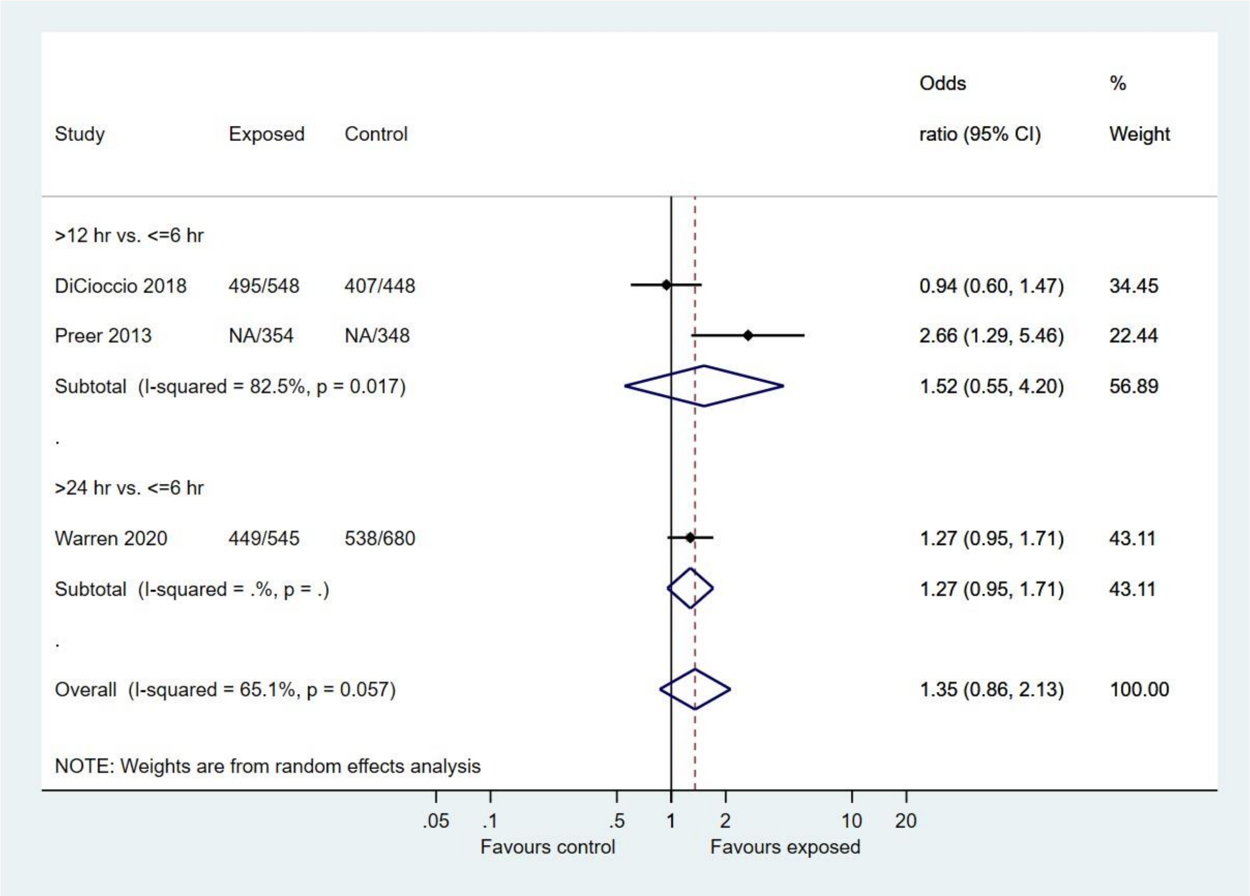

Figure S3. Forest plot for post-hoc analysis: Delayed first bath (>6 h, i.e., at or after 12 or 24 h after birth) vs. early bath (≤6 h after birth) in term, healthy newborns. Outcome: Incidence of breastfeeding initiation
